# *In Vitro* Antimicrobial Activity Evaluation of a Novel Fitostimoline^®^ Plus Spray Formulation

**DOI:** 10.1155/2021/1114853

**Published:** 2021-09-02

**Authors:** Maria Domenica Falciglia, Roberta Palladino, Barbara Maglione, Giulia Schiavo

**Affiliations:** Farmaceutici Damor S.p.a, Via E. Scaglione 27, Napoli, Italy

## Abstract

Wound contaminants are the main cause of healing delay and infection in both chronic and acute wounds; for this reason, the microbial infection management in wound healing is one of the most important components for an effective standard of care. The wound contaminants are most likely to originate from the environment and from the surrounding skin lesion, and to date, the most frequent bacteria isolated are *Staphylococcus aureus*, *Pseudomonas aeruginosa*, and *Klebsiella pneumoniae*. In order to counteract and control these contaminants, the standard care includes topical antiseptic agents. The most commonly used include iodine-releasing agents (e.g., povidone-iodine), hydrogen peroxide, and polyhexanide. This study aims to investigate the *in vitro* antibacterial activity of a novel topical spray (Fitostimoline^®^ Plus spray) based on 0.1% polyhexanide and Rigenase^®^ against *S. aureus*, *P. aeruginosa*, *K. pneumoniae*, and the combination of *S. aureus* and *K. pneumoniae*. The *in vitro* antimicrobial activity of Fitostimoline^®^ Plus spray was evaluated by the agar disk diffusion assay, quantitative suspension test, and quantitative carrier test, demonstrating that Fitostimoline^®^ Plus spray is able to kill 99.9% bacteria. These results support the microbiological characterization of Fitostimoline^®^ Plus spray confirming the antibacterial activity of polyhexanide (PHMB).

## 1. Introduction

Wound contaminants are most likely to originate from the environment (i.e., exogenous microorganisms in the air) and/or from the surrounding skin lesion, involving elements of the normal skin microflora such as *Staphylococcus epidermidis* and *Micrococci*, or from the mucosa membranes [1].

To date, the effect of microorganisms on wound healing has been widely studied, and even if the majority of wounds are polymicrobial, involving both aerobes and anaerobes, the aerobic pathogens (i.e., *S. aureus, P. aeruginosa,* and *K. pneumoniae*) have been most frequently cited as the main cause of delayed wound healing and infection [2].

*S. aureus* is considered the most problematic bacterium in traumatic, surgical, and burn infections [3]. Moreover, *S. aureus* and *K. pneumoniae* are known as the principal responsible for the biofilm formation, a major virulence factor contributing to the chronicity of infections and healing delay. Wounds that exhibit localized signs of infection or that are failing to heal without clinical signs of infection may initially be treated with topical antiseptic agents. An antiseptic strategy has to reach the goal to control/avoid bacteria proliferation and especially has to avoid to create multidrug-resistant organisms (MDROs).

In fact, antibiotics' local application for confined wound infection has to be avoided not only for the risk of promoting drug resistance but also for their microbiostatic mode of action that may result in nonefficacious healing [4].

The topical antiseptic agents most commonly used include iodine-releasing agents (e.g., povidone-iodine and cadexomer iodine), chlorine-releasing solutions (e.g., Dakin's solution and sodium hypochlorite solution), hydrogen peroxide, silver-releasing agents, polyhexamethylene biguanide (PHMB), and acetic acid [3].

Polyhexamethylene biguanide (PHMB) is a synthetic polymer structurally similar to the naturally occurring antimicrobial peptides (AMPs). The structural similarities between AMP and PHMB suggest that the latter can enter the bacterial membrane cells and kill the bacteria in a similar way to AMP [5] through a mechanism of action of adherence and destroying the target cell membranes, causing potassium ions and other cytosolic components leak, resulting in the bacterial cell death [6].

A novel formulation based on 0.1% PHMB and Rigenase^®^ (Fitostimoline^®^ Plus spray) has recently been developed, and in line with the aforementioned issues, we decided to perform this study with the aim to investigate the *in vitro* antibacterial activity of Fitostimoline^®^ Plus spray against the principal microorganisms responsible for wound contamination: S*. aureus, P. aeruginosa, K. pneumoniae*, and the combination of *S. aureus* and *K. pneumoniae*.

## 2. Materials and Methods

### 2.1. Fitostimoline^®^ Plus Spray

Fitostimoline^®^ Plus spray is a medical device for dermatological use contained in a pressurized aluminum bottle. Each pressurized bottle contains Rigenase^®^, 0.1% PHMB, hydroxyethyl cellulose, hydroxypropyl guar hydroxypropyltrimonium chloride, citric acid anhydrous, ethylene glycol monophenyl ether, and purified water. The propellant is dimethyl ether.

### 2.2. Agar Disk Diffusion Assay

Each microorganism (*S. aureus* ATCC 6538 and *P. aeruginosa* ATCC 9027-Microbiologics; *K*. *pneumoniae* ATCC 13883-Thermo Scientific) was streaked onto TSA 90 mm plates (Tryptic Soy Agar-Merck-prepared according to the manufacturer's instructions) for 18–24 h at 30–35°C. Subsequently, one isolated colony was picked from the plate and suspended in NaCl 0.9% and adjusted to equal turbidity of 0.5 McFarland standard. This suspension was used to seed a molten Mueller–Hinton agar medium (Sigma-Aldrich-prepared according to the manufacturer's instructions) stabilized at 45°C (0.1 ml microorganism test suspension/100 ml medium). The obtained cultured medium was poured into 90 mm Petri dishes (20 ml medium/dish) and allowed to solidify. Then, filter paper discs (about 10 mm in diameter) containing the test compounds (Fitostimoline^®^ spray as the negative control and hydrogen peroxide as the positive control—Fitostimoline^®^ Plus spray compared to two different solutions based one on quaternary ammonium salt and the other on povidone-iodine) were placed on the agar surface. The plates were preincubated for 1 h at room temperature to ensure adequate diffusion and finally incubated at 30–35°C for 24 h. The experiments were run in triplicate, and the areas of inhibition were determined with a caliber and recorded as the mean ± SD (*n* = 3) [7, 8].

#### 2.2.1. Statistical Analysis for the Agar Disk Diffusion Assay

Results from the agar disk diffusion assay are expressed as the mean ± standard deviation of three independent experiments performed in triplicate. One-way analysis of variance (ANOVA) was used to test by calculation of *P* values for significant antiseptic activity in bacteria treated with 0.1% polyhexanide topical spray compared to quaternary ammonium salt (QAS) solution and povidone-iodine (PVP-I) solution.

### 2.3. Quantitative Suspension Test

Suspensions of microorganisms (*S. aureus* ATCC 6538, *P. aeruginosa* ATCC 9027, and *K*. *pneumoniae* ATCC 13883) were prepared from one picked isolated colony in NaCl 0.9% and adjusted to equal turbidity of 0.5 McFarland standard. The combination of *S. aureus* and *K. pneumoniae* suspension was prepared by mixing equal amounts of each single suspension prepared as mentioned above.

The suspension test was conducted at room temperature to simulate medical conditions, adding 0.5 ml of the interfering substance (sterile fetal bovine serum-VWR) to 4.5 ml microorganism suspension (10^8^–10^9^ CFU/ml) prior to the addition of 0.75 ml or 1.00 ml (two different doses were performed) of Fitostimoline^®^ Plus spray.

Fitostimoline^®^ Plus spray was added directly to the test microorganisms in a suspension. Sterile neutralizer (3.6 g/l potassium dihydrogen phosphate; 7.2 g/l disodium hydrogen phosphate dihydrate; 4.3 g/l sodium chloride; 1 g/l peptone; 30 g/l Tween 80; 30 g/l saponin; 0.5 g/l histidine; 3 g/l lecithin; 5 g/l sodium thiosulfate pentahydrate) was added after the contact time (15 minutes and 60 minutes) to stop the PHMB effect, the number of surviving bacteria was counted on the agar plate (tryptic soy agar (Merck) prepared according to the manufacturer's instructions by adding 5 g/l Tween 80, 0.7 g/l lecithin, and 1 g/l histidine), and the log reduction was calculated.

The appropriate controls such as media sterilization control, viability growth control, and neutralization control were performed.

According to EN 13727 [9], a product must be able to achieve 5-log reduction (=kill 99.999% bacteria) against the respective test microorganisms to meet the requirements of the European standard.

### 2.4. Quantitative Carrier Test

Each culture of *S. aureus, P. aeruginosa*, and *K. pneumoniae* was initiated from frozen stock in tubes containing 10 ml of tryptic soy broth (Merck). After a 24-hour incubation at 30–35°C, 1 ml of the suspension was transferred to 9 ml of fresh tryptic soy broth incubated for 48–54 h to obtain 10^8^–10^9^ CFU/ml broth culture, and 0.1 ml aliquot of FBS (interfering organic soil) was added to 1.9 ml of each broth culture (the combination of *S. aureus* and *K. pneumoniae* suspension was prepared by mixing an equal amount of each single broth culture prepared as mentioned above). Carriers were prepared by placing autoclaved 25.4 × 76.2 mm glass slides into sterile Petri dishes with 2/3 sheets of paper. One loop of the test suspension was spread over the whole glass surface and dried for 20 minutes at 30–35°C, and each glass carrier should contain >10^5^ CFU/carrier. Ten test carriers for each suspension test were sprayed (one pump for three seconds) individually from a distance of 10–15 cm at a 45° angle. Control carriers were treated with 1 ml of PBS. After contact times of 15 minutes ±5 seconds and 60 minutes ±5 seconds, each carrier was neutralized with 20 ml of the neutralizer (3.6 g/l potassium dihydrogen phosphate; 7.2 g/l disodium hydrogen phosphate dihydrate; 4.3 g/l sodium chloride; 1 g/l peptone; 30 g/l Tween 80; 30 g/l saponin; 0.5 g/l histidine; 3 g/l lecithin; 5 g/l sodium thiosulfate pentahydrate). Using standard surface spread techniques, the neutralized substance was plated on tryptic soy agar (prepared according to the manufacturer's instructions by adding 5 g/l Tween 80, 0.7 g/l lecithin, and 1 g/l histidine) and incubated for 24 h at 30–35°C. The surviving microbial population on the test plates was compared to the controls to determine log reduction. The ASTM (American Society for Testing and Materials) E1153 [10, 11] passing criterion is >3 log_10_ reduction (=kill 99.9% bacteria). Appropriate controls were performed.

## 3. Results

### 3.1. Agar Disk Diffusion Assay

Agar diffusion test is a well-standardized, simple test, useful for the evaluation of the susceptibility of the isolate and diffusion rate of the drug through the agar medium. It is considered as a qualitative test to investigate the antimicrobial activity of a substance or, as in this case, of a novel product. Agar disk diffusion assay was used to evaluate the antimicrobial efficacy of Fitostimoline^®^ Plus spray compared to two other products for which antimicrobial and disinfectant activities were already demonstrated. H_2_O_2_ was used as a positive control, and Fitostimoline^®^ spray was used as a negative control to confirm that the supposed antimicrobial activity of Fitostimoline^®^ Plus spray was due to the addition of 0.1% PHMB in formulation. The results are summarized in Table 1.

The mechanism of action of QASs can be summarized as follows. In the first stage, the QAS molecule adsorbs on the cell wall and penetrates it [12]. Further activity assumes the reaction with lipids and proteins of the cell membrane, which leads to disorganization in its structure and the leakage of low-molecular components out of the cell. Then, proteins and nucleic acids degrade inside the cell. The release of autolytic enzymes leads to the lysis of the cell wall components. The observable effect of these processes is a complete loss of the structural organization of the cell [12, 13].

PVP-I is a water-soluble iodophor (or iodine-releasing agent) that consists of a complex between iodine and a solubilizing polymer carrier, polyvinylpyrrolidone [10, 11]. In aqueous solution, a dynamic equilibrium occurs between free iodine (I2), the active bactericidal agent, and the PVP-I-complex. As a small molecule, iodine rapidly penetrates into microorganisms and oxidizes key proteins, nucleotides, and fatty acids, eventually leading to cell death [14, 15]. PVP-I has a broad antimicrobial spectrum with activity against Gram-positive and Gram-negative bacteria, and its action against *S. aureus* is well documented [16].

**Figure 1:**
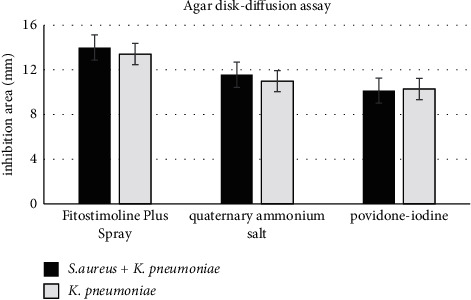
Agar disk diffusion assay inhibition areas against *K. pneumoniae* and a mix of *K. pneumoniae* and *S. aureus* of Fitostimoline^®^ Plus spray compared with the ones of a quaternary ammonium salt solution and povidone-iodine solution. Results are the mean ± standard deviation of three independent experiments. *P* values are calculated by the one-way ANOVA test (*P* < 0.01 Fitostimoline^®^ Plus spray vs. each of the others).

According to literature data, PVP-I and QAS have a good susceptibility against *S. aureus* (Gram-positive bacteria), but also, Fitostimoline^®^ Plus spray provides an inhibition zone diameter even if it is not statistically significant. Instead, Figure 1 shows that agar diffusion assay inhibition areas of Fitostimoline^®^ Plus spray against *K. pneumoniae* (Gram-negative bacteria) and against a mixture of *K. pneumoniae* + *S. aureus* are statistically significant compared with the ones provided by a quaternary ammonium salt solution and a povidone-iodine solution (according *P* values calculated by the one-way ANOVA test—*P* < 0.01 Fitostimoline^®^ Plus spray vs. each of the other evaluated products). It is particularly interesting that the lack of inhibition zone diameter shown by the three products, Fitostimoline^®^ Plus spray, PVP-I, and QAS solutions, against *P. aeruginosa* demonstrates the limit of the agar disk diffusion assay due to the insufficient amount of the product in the agar disk.

### 3.2. Quantitative Suspension Test

**Figure 2:**
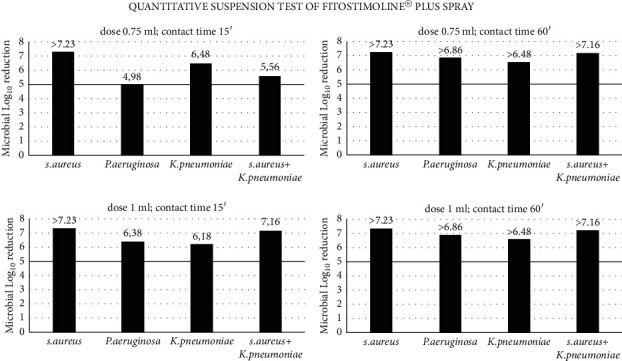
Antimicrobial activity of two doses of Fitostimoline^®^ Plus spray for two different contact times 15' and 60' against microbial suspension in the presence of organic material (FBS) of *S. aureus*, *P. aeruginosa*, *K. pneumoniae*, and a mix of *S. aureus* and *K. pneumoniae*. The results are expressed as microbial log10 reduction measured in terms of remaining alive colony-forming units (CFUs) after the product's action. The horizontal black limit line indicates 99.999% bacteria killing.

Interfering substance was used to simulate organic materials present in wounds, which can affect the product's antimicrobial efficacy. A suspension of a great number of microorganisms is directly in contact with a dose of Fitostimoline^®^ Plus spray.  Table 2 and Figure 2 show the results of a quantitative suspension test in which two different doses of Fitostimoline^®^ Plus spray were tested against *S. aureus, P. aeruginosa, K. pneumoniae*, and a mixture of *S. aureus* *+* *K. pneumoniae* at two different contact times, 15 minutes and 60 minutes. To pass the standard performance criteria for successful antimicrobial efficacy, the product must kill 99.999% bacteria. 1.00 ml dose of Fitostimoline^®^ Plus spray at both contact times successfully met the efficacy performance criteria. The results show that only for 0.75 ml dose and *P. aeruginosa* at contact time 15 minutes, the product did not meet the efficacy criteria even if log_10_ reduction is 4.98 and not 5, but after 60 minutes, the same dose, even for *P. aeruginosa*, is able to achieve > 6 log_10_ reduction. These results prove an antimicrobial efficacy of 0.75 ml of Fitostimoline^®^ Plus spray after a contact time of 60 minutes.

### 3.3. Quantitative Carrier Test

Carrier test mimics the actual conditions better than the suspension test because microorganisms are predried and adhered to glass simulating actual wound conditions such as contact time, temperature, and soiling.

According to the flow analysis of the spray nozzle for a distance of 10–15 cm (0.35 g/ml) and the product density (0.95 ÷ 1.1 g/ml), one pump for 3 seconds similar to 1.2 g or 1 ml of the product is considered.

**Figure 3:**
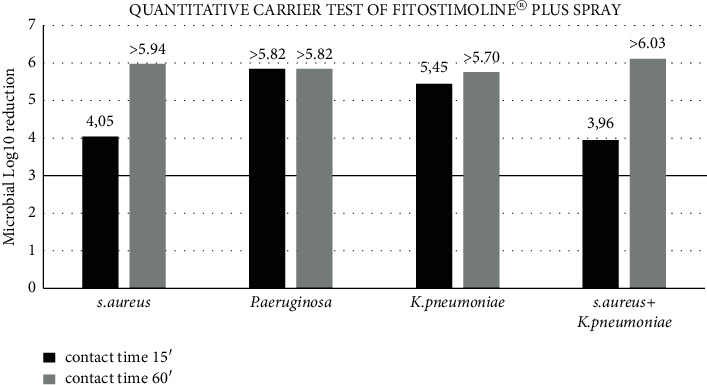
Antimicrobial activity of one pump (duration: 3 seconds) of Fitostimoline^®^ Plus spray for two different contact times (15 and 60 minutes) against *S. aureus*, *P. aeruginosa*, *K. pneumoniae*, and a mix of *S. aureus* and *K. pneumoniae* on glass carriers in the presence of FBS. The results are expressed as microbial log10 reduction measured in terms of remaining colony-forming units (CFUs) on the glass carrier after the product's action. The horizontal black limit line indicates 99.9% bacteria killing.

Table 3 and Figure 3 show the results of the quantitative glass carrier in which one pump for 3 seconds of Fitostimoline^®^ Plus spray was tested against *S. aureus, P. aeruginosa, K. pneumoniae*, and a mixture of *S. aureus* *+* *K. pneumoniae* at two different contact times, 15 minutes and 60 minutes. To pass the standard performance criteria for successful antimicrobial efficacy, the product must kill 99.9% bacteria. After a contact time of 15 minutes, some glasses show the growth of bacteria, except *P. aeruginosa*; instead, after a contact time of 60 minutes, Fitostimoline^®^ Plus spray kills more than 99.999% bacteria, achieving more than 3 log_10_ reduction.

## 4. Discussion and Conclusion

There is general consent that microbial load needs to be kept under control in skin lesions in order to ensure effective wound healing [17].

With this aim, Farmaceutici Damor has developed Fitostimoline^®^ Plus spray, a novel topical medical device, which, thanks to its particular formulation based on Rigenase^®^, forms a protective barrier against the external environment, creating favorable conditions for a rapid and correct re-epithelizing action on the skin, and, due to PHMB, helps to keep the microenvironment and contamination under control.

Rigenase^®^, a specific patented *Triticum vulgare* extract, exhibits antioxidant capacity by tissue-repairing activity and moisturizing action [18]; thus, it is used in the formulation of different medical devices—under the brand name Fitostimoline^®^—with properties of accelerating tissue repair, improving the restoration of the epidermal barrier, and stimulating lipid synthesis [19].

The antiseptic agent polyhexamethylene biguanide (also known as polyhexanide or PHMB) can penetrate bacterial cell membranes and kill bacteria simulating antimicrobial peptides (AMPs) produced by many cells within the wound [5, 20]. Studies have shown that PHMB has a broad spectrum of activities both *in vitro* and *in vivo*, and it is safe for clinical use. Polyhexanide topical preparation was confirmed to be at least as efficacious as silver sulfadiazine in the treatment of microbial infections, supporting the use of PHMB for the treatment of wounds that are infected or at risk of infection [21].

This study's first investigation was to detect a new topical spray's antimicrobial activity, comparing its action with two commonly used wound antiseptics, using the agar disk diffusion assay. The results showed a similar antimicrobial activity among the products even if it is depending on the dose; however, in the case of *K. pneumoniae* and a mix of *S. aureus* and *K. pneumoniae*, as shown in Figure 1, Fitostimoline^®^ Plus spray seems to be more effective than PVP-I and QAS solutions.

The suspension test was ideal to define the dose and contact time to test the Fitostimoline^®^ Plus spray biocidal action. As we assumed that Fitostimoline^®^ Plus spray application is indicated for wound treatment, a large amount of bacteria was mixed with protein and organic load (10% FBS which potentially inhibits the product's antimicrobial efficacy), and the suspension was exposed to a defined amount of Fitostimoline^®^ Plus spray for a specific contact time. After neutralization, the number of survivors was estimated. Two different doses and two different contact times were tested. Dose 1.00 ml (corresponding to 1 pump for 3 seconds in spray) is more effective than dose 0.75 ml already after a contact time of 15 minutes.

Suspension test was performed to reflect clinical conditions to ensure that 1 pump for 3 seconds of Fitostimoline^®^ Plus spray is able to inactivate microorganisms, and its efficacy was tested under conditions close to real life, with a quantitative carrier test using glass as the carrier and FBS as the organic soil for two contact times, 15 and 60 minutes. The results of this study show that 1 pump for 3 seconds of Fitostimoline^®^ Plus spray exerts antimicrobial activity, killing more than 99.9% of *S. aureus, P. aeruginosa, K. pneumoniae*, and a mixture of *S. aureus* *+* *K. pneumoniae* at room temperature after a contact time of 15 minutes according to ASTM E1153 [13].

The obtained results lead us to the conclusion that Fitostimoline^®^ Plus spray, other than its regenerating activity, has an antimicrobial activity related to the presence of PHMB in the formulation.

## Figures and Tables

**Table 1:** Summary of agar disk diffusion assay inhibition areas (mm).

	Fitostimoline^®^ Plus spray	Quaternary ammonium salt solution	Povidone-iodine solution	Positive control	Negative control
*S. aureus*	13.71 ± 1.16	20.00 ± 1.77	19.29 ± 3.24	32.57 ± 2.72	0.00 ± 0.00
*P. aeruginosa*	0.00 ± 0.00	0.00 ± 0.00	0.00 ± 0.00	27.85 ± 1.88	0.00 ± 0.00
*K. pneumoniae*	13.43 ± 0.49	11.00 ± 0.76	10.29 ± 0.45	27.86 ± 1.88	0.00 ± 0.00
*S. aureus* + *K. pneumoniae*	14.00 ± 0.93	11.57 ± 0.73	10.14 ± 0.35	28.00 ± 3.21	0.00 ± 0.00

Results are the mean ± standard deviation of three independent experiments.

**Table 2:** Summary of the microbial log_10_ reduction and percentage after the action of Fitostimoline^®^ Plus spray.

Microorganism	Final inoculum size (CFU/5 ml)	Dose 0.75 ml				Dose 1.00 ml			
		Contact time				Contact time			
		15'		60'		15'		60'	
		Reduction		Reduction		Reduction		Reduction	
		Log_10_	%	Log_10_	%	Log_10_	%	Log_10_	%
*S. aureus*	1.70*E *+* *08	>7.23	99.99999	>7.23	99.99999	>7.23	99.99999	>7.23	99.99999
*P. aeruginosa*	7.30*E *+* *07	4.98	99.99895	>6.86	99.99999	6.38	99.99996	>6.86	99.99999
*K. pneumoniae*	3.00*E *+* *07	6.48	99.99997	>6.48	99.99997	6.18	99.99993	>6.48	99.99997
*S. aureus + K. pneumoniae*	1.43*E *+* *08	5.56	99.99972	>7.16	99.99999	7.16	99.99999	>7.16	99.99999

**Table 3:** Summary of the microbial log_10_ reduction and percentage after the action of Fitostimoline^®^ Plus spray on a contaminated glass surface.

Microorganism	Contact time: 15'				Contact time: 60'			
	Initial counts on the glass surface after contact time (CFU/glass)	Number of treated carriers showing growth	Reduction		Initial counts on the glass surface after contact time (CFU/glass)	Number of treated carriers showing growth	Reduction	
			Log_10_	%			Log_10_	%
*S. aureus*	1.39*E* + 06	10/10	4.05	99.99109	1.72*E* + 06	0/10	>5.94	99.99989
*P. aeruginosa*	7.10*E* + 05	0/10	>5.82	99.99985	7.10*E* + 05	0/10	>5.82	99.99985
*K. pneumoniae*	5.50*E* + 06	5/10	5.45	99.99965	1.01*E* + 06	0/10	>5.70	99.99980
*S. aureus* *+* *K. pneumoniae*	1.12*E* + 06	10/10	3.96	99.98904	2.11*E* + 06	0/10	>6.03	99.99991

## Data Availability

The data that support the findings of this study are available from the corresponding author upon reasonable request.
